# Alternative Protein and Iron Sources from Edible Insects but Not *Solanum torvum* Improved Body Composition and Iron Status in Malnourished Rats

**DOI:** 10.3390/nu11102481

**Published:** 2019-10-16

**Authors:** Isaac Agbemafle, Nicole Hanson, Amanda E. Bries, Manju B. Reddy

**Affiliations:** 1 Food Science and Human Nutrition, Iowa State University, 2302 Osborn Dr, 220 Mackay, Ames, IA 50011, USA; iagbemafle@gmail.com (I.A.); nchanson@iastate.edu (N.H.); aebries@iastate.edu (A.E.B.); 2 School of Public Health, University of Health and Allied Sciences, Hohoe PMB 31, Volta Region, Ghana

**Keywords:** iron bioavailability, crickets, palm weevil larvae, turkey berry, protein status

## Abstract

*Solanum torvum* (STO) and edible insects are potential dietary approaches to prevent malnutrition. Hence, we determined the effect of STO and insect powders on improving nutritional status in malnourished rats. Malnutrition was induced in rats by feeding 5% protein, ~2 ppm Fe (LPI) diet for 21 days. During the 14 day repletion, five groups of rats (*n* = 8) were fed diets supplemented with *Acheta domesticus* (cricket, ADO), *Rhynchophorus phoenicis fabricius* (palm weevil larvae, RFA), STO, ADO + STO (TAD), and casein + ferrous sulfate (PIS, positive control), as well as a non-supplemented group (negative control, LPI). A normal (NOM) group was fed protein-Fe sufficient (PIS) diet throughout the study. Body composition was measured by Dual-energy X-ray absorptiometry. The hemoglobin (Hb) repletion method was used to assess relative biological value (RBV, compared to PIS) of the supplemented groups. No differences were found in weight gain, bone mineral content, lean and fat mass, and organ weights among the edible insects and PIS groups, but these results differed from STO and the LPI groups. An increase in Hb Fe and RBV with ADO and RFA was comparable to PIS. ADO and RFA could be excellent sources of protein and bioavailable Fe, making it a sustainable, low-cost food source to prevent malnutrition in humans.

## 1. Introduction

Protein is essential for growth and development, and iron (Fe) is an essential micronutrient for hemoglobin (Hb) synthesis. Low intakes of protein and bioavailable Fe results in protein-energy malnutrition (PEM) and Fe-deficiency anemia (IDA), which are common in developing countries. Despite some progress being made from 1990 to 2017, PEM and IDA prevalence remain unacceptably high worldwide. New evidence indicates that at least 151 million children (22%) in 2017 experienced stunted growth (low height-for-age) compared to 165 million children (26%) in 2011 [1,2]. These global estimates are highest in South Asia and sub-Saharan Africa. Globally, wasting (low weight-for-height) and childhood obesity continues to affect over 50 million and at least 38 million children under 5 years of age [1]. Anemia prevalence decreased in children from 47% in 1995 to 43% in 2011 [3]. The slow progress made in reducing undernutrition (stunting, wasting, and anemia) has important consequences for child survival, infectious and chronic disease burden, cognitive and motor development as well as economic productivity [2,3,4]. For example, in children under 5 years, stunting and wasting contributes to 14.5%, and 14.6% of global deaths and a further 3.1 million deaths are attributed to micronutrient deficiencies [2,4]. These statistics are a clear indication of the need to scale-up nutrition-sensitive interventions to ensure no child is left behind in achieving the Sustainable Development Goals (SDGs) on food and nutrition security as enshrined in Agenda 2030. 

Addressing the challenges of hunger, food insecurity, and malnutrition is prominently laid out in goal 2 of the SDGs. These goals provide a compelling new way of thinking to reduce the cost of nutritious foods and the stress of living with food insecurity. Due to their cheap, sustainable, and high availability, insects are a favorable food source. Therefore, it is not surprising that since 2013, the Food and Agriculture Organization (FAO) has enhanced its efforts in promoting increased consumption of insects and other underutilized crops as a strategy to alleviate food and nutrition security by 2025 [5]. These underutilized foods increase food and nutrition security through improved dietary diversity and quality and are increasingly recognized as a cost-effective and sustainable way of improving malnutrition [6]. However, due to the lack of attention on underutilized foods, significant gaps exist in our current knowledge of their contribution to human nutrition. 

Among these underutilized foods, *Solanum torvum* (STO, commonly known as turkey berry), *Rhynchophorus phoenicis fabricius* (RFA, widely known as palm weevil larvae) and *Acheta domesticus* (ADO, commonly known as house cricket) are of interest to study. Cricket and palm weevil larvae have comparable protein biological value to meat and fish and even significantly higher levels of iron than beef [7,8]. Also, in tropical and subtropical countries, turkey berry is known to be a rich source of iron, among other micronutrients [9]. Turkey berry was chosen among other plants because it grows wild and it is well studied for its antioxidant properties, as well as its traditional use as a hematinic plant. These underutilized foods offer a great potential [10] in developing countries and are nowadays gradually “rediscovered” for human consumption, although the evidence for its contribution to reducing malnutrition is lacking. Thus, understanding the bioavailability of nutrients in these underutilized foods is crucial to determine the specific qualitative recommendations for their use. Hence, the objective of this study was to determine the protein and Fe bioavailability of these underutilized foods relative to a casein and ferrous sulfate (FeSO_4_·7H_2_O) diet, using the hemoglobin/protein repletion method in rats. 

## 2. Materials and Methods 

### 2.1. Food Samples and Chemicals

Fresh unripe fruits of STO were purchased from Adawso market in Mampong in the Eastern region, Ghana. The fruits were washed with distilled water, crushed with a mortar and pestle, and dried using a locally manufactured solar dryer at the Center for Plant Medicine Research in Mampong, Eastern Region, Ghana. Solar drying lasted for 5–7 days at 70 °C. A hammer mill (Bravo Grinder, Washington, IA, USA) was used to grind the samples and they were stored in labeled Ziploc bags. The samples were transported by air to the United States. Fresh palm weevil larvae samples were harvested from Aspire Food groups center in Kumasi in the Ashanti region, Ghana, and they were washed with distilled water to remove feed particles. The larvae were then soaked in a clean container of distilled water for 15 minutes to allow them to clear their gut. Larvae were recleaned and blanched in hot water at 95 °C for 2 minutes. Inactivated larvae were then milled with a small amount of water in a blender (Hamilton beach, Southern Pines, NC, USA) at top speed for 5 minutes until a smooth slurry was obtained. The samples were stored in labeled Ziploc bags, frozen for 24 h, then transported on ice to the United States for further processing. When samples were received in the United States, they were kept at −20 °C for short term storage for 1 week. Samples were then spread on trays and kept at −80 °C for about 24 h prior to freeze-drying. Subsequently, samples were dried for 24–48 h using a freeze dryer (Hudson Valley Lyomac Trilogy Freeze dryer, Hudson, NY, USA). The freeze-dried samples were labeled and stored in Ziploc bags at −20 °C. Ready to use powdered samples of cricket flour were obtained from Aspire Food Groups, Austin, Texas, USA. Briefly, the crickets were frozen to inactive form, and during processing, they were thawed, rinsed, and wet-milled to a fine grind. The slurry of ground crickets was pasteurized at 68 °C for 35 min before drying into powder form. Ferrous sulfate and all other chemicals used for laboratory analysis were analytical grade obtained from Sigma-Aldrich, St. Louis, MO, USA. 

### 2.2. Animals and Diet 

**Figure 1:**
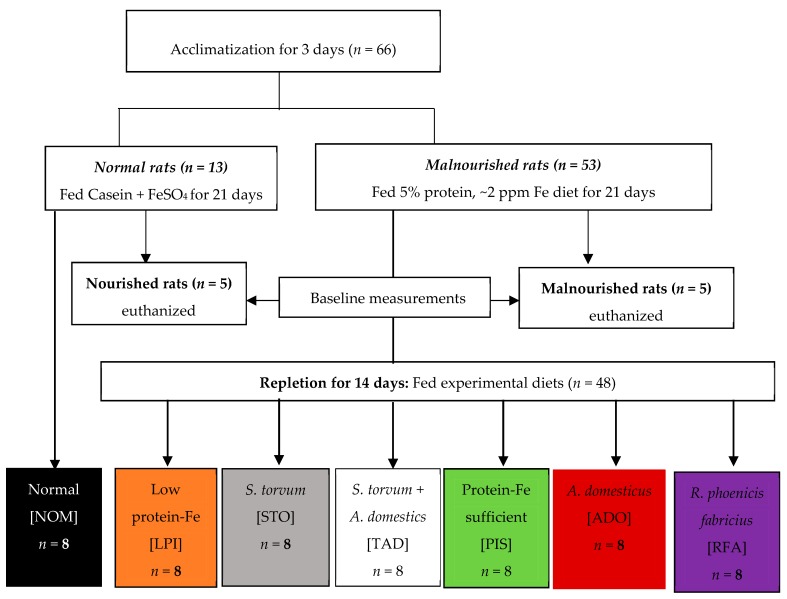
Study Flow Diagram.

Male weanling Sprague–Dawley rats (*n* = 66; Charles River, Chicago, IL, USA) at 21 days of age with an initial body of ~70 g were housed individually in innovive cages for the first 25 days and then placed in conventional cages with wire bottoms for the remaining 14 days of the study. Rats were kept under controlled conditions with a daily 12 h light: dark cycle. Distilled water and diet were fed ad libitum. Rats were acclimated for 3–4 days on standard rat chow and then randomized into the normal (*n* = 13) and malnourished (*n* = 53) group, as shown in Figure 1.

Subsequently, the healthy group was divided into one group (nourished) of five rats and another group (normal, NOM) of eight rats. The malnourished group was further divided into six groups of eight rats and one group of five. Rats in the normal group consumed 15% protein from casein and 20 ppm Fe from ferrous sulfate; this diet is herein referred to as protein-Fe sufficient (PIS). Rats in the malnourished group consumed 5% protein and ~2 ppm Fe; this diet is herein referred to as low protein-Fe (LPI). Rats in the normal and malnourished groups were fed ad libitum for 21 days (Figure 1). Subsequently, four malnourished groups (*n* = 8 each) were repleted for 14 days with the LPI diet supplemented with STO, ADO, RFA, and cricket + turkey berry (TAD). One malnourished group was switched to the PIS diet (positive control) while another group remained on the LPI diet (negative control). The eight rats from the normal group are herein referred to as NOM group remained on the PIS diet throughout the study. The other five rats in the nourished group and the five in the malnourished group were euthanized after the 21 day feeding for baseline measurements. All repletion diets contained 20 ppm Fe, but the RFA diet contained 10 ppm Fe because of its low Fe content. Also, the repletion diets were matched for protein (15%), except for the STO diet (8.8%) and ADO diet (23.3%) because of their low and high protein content, respectively. All diets were isocaloric (3.9 kcal/g) except the RFA diet, which was (4.3 kcal/g) due to the high-fat content of the larvae. Diets, as prepared by Envigo Tekland (Madison, WI, USA), are shown in Table 1. The nutrient composition of the diets was verified by MidWest Laboratories (Omaha, NE, USA). All procedures involving animals were reviewed and approved by the Institutional Animal Care and Use Committee (IACUC ID: 11-17-8648-R) at Iowa State University, Ames, Iowa, USA.

### 2.3. Growth Assessment and Tissue Collection 

During the 21 day feeding period, rats were weighed every 3–4 days, and food intake was recorded every 3 days. On day 21, five rats in the nourished and malnourished group were fasted for 12 h and euthanized between 8:00–12:00 h for baseline measurements to compare nourished and malnourished groups. The remaining malnourished rats were weighed, fasted for 12 h, and blood collected by tail bleeding for Hb determination. Under anesthesia, a small cut was made 1 mm from the tip of the tail using a scalpel blade, and blood was collected into the hemocue cuvette for Hb reading. Subsequently, the rats began the 14 day repletion period, during which food intake and body weight was recorded every other day. 

At the end of the repletion period, rats were weighed and anesthetized via an intraperitoneal injection of ketamine:xylazine (90:10 mg/dL). Blood was collected via cardiac puncture, and subsequently, the spleen, right kidney, and liver were removed and weighed. The brain was also removed (spinal cord separated at the caudal extent of the cerebellum) from the skull and weighed to the nearest 0.01g. 

### 2.4. Determination of Body Composition Using Dual-Energy X-Ray Absorptiometry (DXA)

The fresh carcasses were carefully positioned individually on an absorbent paper towel in a supine position paying attention to body alignment. The fore- and hind-limbs of the rats were placed perpendicular to the long axis of the body. The tail was positioned in a curve towards the head so that it would lie within the DXA scan field. A small piece of adhesive tape was used to hold the tail, fore- and hind-limbs in place to maintain the rat in this position. The rats were then transferred in flat boxes for body composition analysis using DXA. 

The DXA instrument (QDR 4500W, Hologic, Bedford, MA, USA) with the Hologic QDR Software (for Windows XP version) was used for the rat scans. A quality control procedure was routinely performed with a calibration phantom before imaging. Individual rat measurements were obtained from the scan of four rats at a time. Briefly, whole-body scans were done in the infant mode to provide total and regional body composition measurements. Five minute scans were analyzed with the manufacturer’s software (version 5.7c), and values for body surface area, bone mineral content (BMC), bone mineral density, fat mass (FM), percent FM, lean mass (LM) and total body mass were recorded. 

### 2.5. Determination of Serum Albumin 

The euthanized rats were perfused transcardially with a 5 mL 21 G syringe (BD, Franklin Lakes, NJ; USA) into vacutainer tubes. After the whole blood had clotted between 30–60 mins, it was centrifuged at 12,500 g using a minicentrifuge (horizon mini E, Quest Diagnostics, Urbandale, IA, USA). The serum was stored in labeled microcentrifuge tubes at −80 °C (Ultra-Low Freezer, So-Low Environmental Equipment, Cincinnati, OH, USA) until ready for analysis. On the day of analysis, frozen serum was allowed to reach room temperature. Serum albumin, as a measure of protein status, was determined by a commercial kit based on the Bromocresol green albumin assay (BioAssay Systems, Hayward, CA, USA). 

### 2.6. Determination of Hemoglobin Concentration 

Hemoglobin concentration was measured in the whole blood using hemocue Hb 201+ hemoglobin analyzer (Medical Device Depot Inc., Ellicott City, MD, USA). Total hemoglobin concentration (HbC) was calculated based on 0.075 L of blood/kg body weight and the measured hemoglobin value (g/L). Hemoglobin iron (Hb Fe) was calculated based on blood volume relative to body weight, and the amount of Fe contained in hemoglobin, as shown below [11]:

(1) $$\mathrm{Hb}\;\mathrm{Fe},\;\mathrm{mg}=body\;wt,kg\;\times \frac{0.075\;L\;blood}{\;body\;wt,\;kg}\times \frac{\mathrm{Hb},\;g}{blood,\;L}\times \frac{3.35\;\mathrm{mg}\;\mathrm{Fe}}{Hb,\;g}.$$

Hemoglobin regeneration efficiency (HRE) values were then calculated for each rat as a measure of the efficiency of converting dietary Fe into Hb Fe [11]. Subsequently, relative biological value (RBV) for each iron source compared to ferrous sulfate was calculated as the HRE of test Fe source for each rat divided by the mean HRE of ferrous sulfate (PIS group).

(2) $$\mathrm{HRE}=\frac{{\left(Hb\;Fe,\;mg\right)}_{final}-{\left(Hb\;Fe,\;mg\right)}_{initial}}{Fe\;consumed,\;mg}$$

### 2.7. Statistical Analysis 

Differences in body weight (BW) were calculated separately for the 21 day feeding and 14 day repletion period. Changes in body composition, organ weights, and serum albumin were obtained by subtracting the average value for every measurement in the malnourished group from each of the repleted (LPI, STO, TAD, PIS, ADO, and RFA) groups. Similarly, for the NOM group, changes in body composition, organ weights, and serum albumin were obtained by subtracting the average value for every measurement in the nourished group from each of the NOM rats at the end of the study. Change in Hb concentration was obtained by subtracting Hb at the end of the 21 day feeding period from the end of the 14 day repletion period. 

Data were analyzed using GraphPad Prism version 8 (La Jolla, CA, USA). Data are expressed as mean ± SEM. Statistical differences between baseline malnourished and nourished groups were compared using Student’s *t*-test. Group comparisons for the change in measurements for the different groups were performed using one-way ANOVA followed by Tukey’s multiple comparison tests with significance set at *p* < 0.05. 

## 3. Results

The level of dietary protein and Fe content of the diets (Table 1) had a significant effect on daily food intake during the 21 day feeding period. Malnutrition decreased weight gain in proportion to the amount of food eaten for the malnourished (164.2 ± 14.5 g) and the nourished groups (313.9 ± 10.1 g) (*p* < 0.002, Table 2). Moreover, food, energy, carbohydrates, fats, and zinc intakes were about 50% lower in the malnourished group compared to the nourished group (*p* < 0.002, Table 2). Protein and iron intakes were about six times higher in the nourished group compared to the malnourished group (*p* < 0.002, Table 2). 

**Figure 2:**
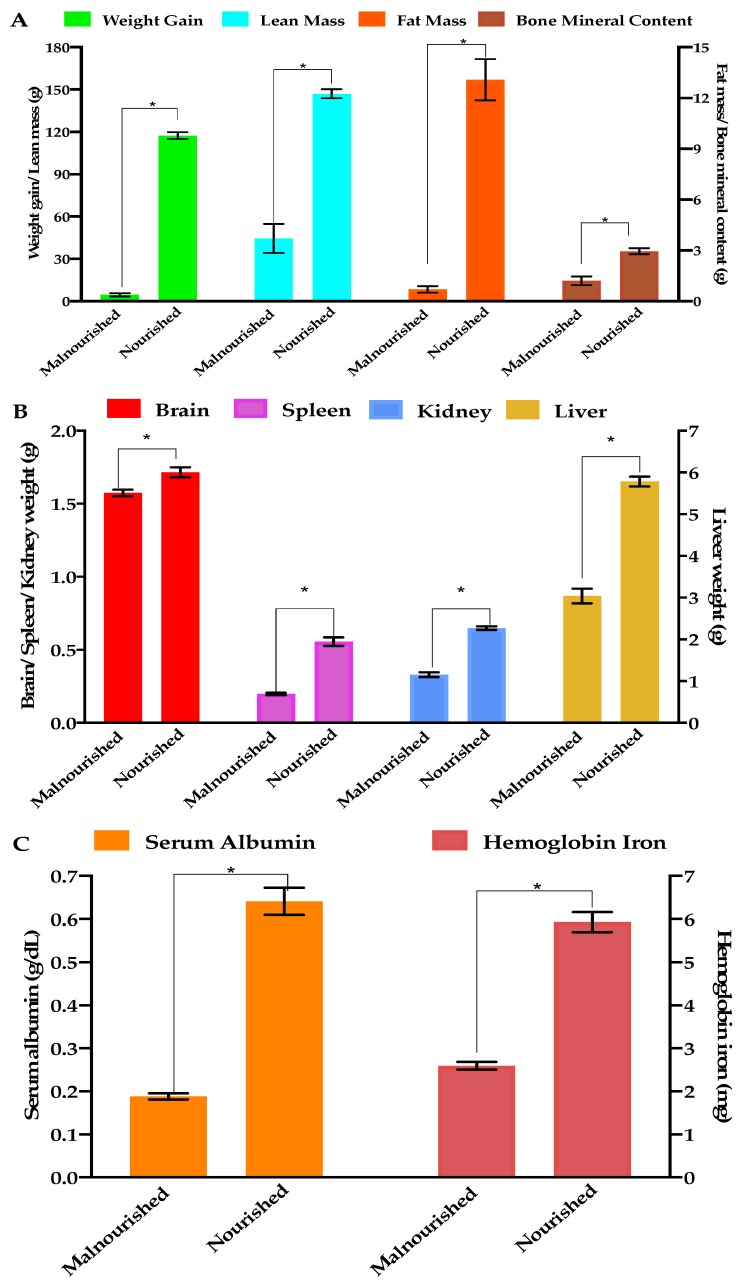
Effects of feeding low protein-Fe and protein-Fe sufficient diets on body composition, organ weights, serum albumin, and hemoglobin iron. (**A**) Weight gain, lean mass, fat mass, bone mineral content, (**B**) Brain, spleen, kidney, liver weights, (**C**) Serum albumin, hemoglobin iron. Data are presented as means ± SEM, *n* = 5, * Significant at *p* < 0.008 according to students’ *t*-test.

At the end of the 21 days, rats in the malnourished group showed a decrease in BW gain (4.6 ± 1.1 g) compared to rats in the nourished group (117.4 ± 2.4 g) (Figure 2A). LM (44.44 vs. 147.06 g), FM (0.70 vs. 13.08 g), and BMC (1.20 vs. 2.96 g) in the malnourished group (Figure 2A) were significantly lower compared to the nourished group. Brain (1.57 vs. 1.72 g), liver (3.04 vs. 5.78 g), spleen (0.20 vs. 0.56 g), and kidney (0.33 vs. 0.65 g) weights were lower in the malnourished group compared to the nourished group (Figure 2B; *p* ≤ 0.008). Serum albumin (0.19 vs. 0.64 g/dL) and Hb Fe (2.59 vs. 5.93 mg) were significantly lower in the malnourished group compared to the nourished group (Figure 2C; *p* < 0.0001). 

Overall, there was no difference in food, and nutrient intake during the 21 day feeding for all the different groups of rats fed the low protein-Fe diet. However, during the 14 day feeding, food (281.9 ± 12.4 g) and energy (1099.4 ± 48.4 kcal) intakes were highest in the NOM group, but this was not significantly different from the food (246.7 ± 11.3 g) and energy (962.1 ± 43.9 kcal) intakes in the TAD group (Table 3). Food (147.7 ± 5.4 g) and energy (576.0 ± 21.0 kcal) intakes were lowest in the LPI group, and this was significantly lower compared to all other groups. There were no differences in food and energy intakes among the STO, PIS, ADO, and RFA groups (Table 3). There were no significant differences in carbohydrate intake among the STO (30.2 ± 1.7 g), TAD (37.0 ± 1.7 g), PIS (33.8 ± 0.9 g), and RFA (31.9 ± 1.2 g) groups. Although protein (113.4 ± 4.1 g) intake was lowest in the LPI group, this was not significantly different among the STO (134.6 ± 7.7 g), ADO (125.9 ± 5.8 g), and RFA (123.0 ± 4.8 g) groups. There were no significant differences in protein intake among the STO, TAD, PIS, and ADO groups. No differences in protein intake were observed among the STO, TAD, ADO, and RFA groups (Table 3). Protein (188.6 ± 8.3 g) intake was significantly higher in the NOM group compared to any other group. However, fat intake in the NOM (19.9 ± 0.9 g) group was not significantly different from the ADO (19.5 ± 0.9 g) group. As expected, fat intake was significantly highest in the RFA (33.6 ± 1.3 g) group and lowest in the STO (14.2 ± 0.8 g) and LPI (10.4 ± 0.4 g) groups. For Fe intake, there were no differences between the NOM (5.6 ± 0.3 mg) and TAD (4.9 ± 0.2 mg) groups. Iron intake was not significantly different among the STO (4.0 ± 0.2 mg), PIS (4.5 ± 0.1 mg), and ADO (4.5 ± 0.2 mg) groups. There were no differences in iron intake among the TAD, PIS, and ADO groups. The iron intake for RFA (2.1 ± 0.1 mg, Table 3) was about 2.5 times lower compared to the STO, TAD, PIS, ADO, and NOM groups (*p* < 0.0001). Zinc intake was highest in the ADO (23.9 ± 0.9 g) group, and this was significantly different from the PIS (20.6 ± 1.0 mg) and NOM (10.0 ± 0.4 mg) groups. Zinc intake in the ADO group was three times the level in the LPI (8.7 ± 0.5 mg) group. There were no significant differences in zinc intake in the NOM, LPI, TAD, and RFA groups, as shown in Table 3. 

**Figure 3:**
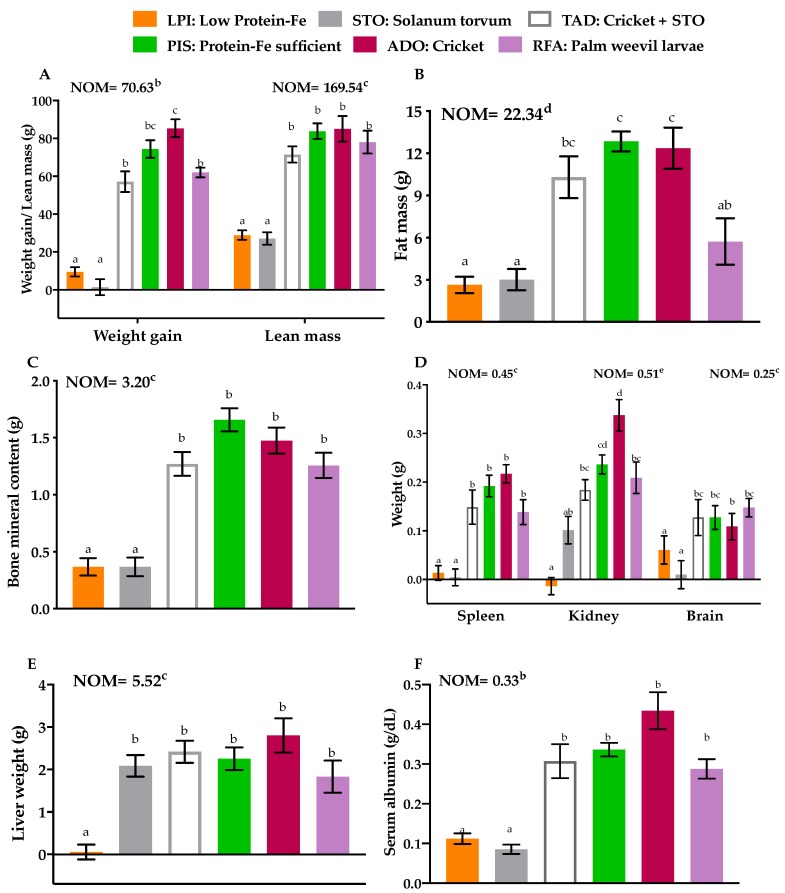
Effects of protein-Fe supplementation on body composition, organ weight, and serum albumin. (**A**) Weight gain, lean mass (**B**) Fat mass (**C**) Bone mineral content (**D**) Brain size, spleen, kidney weights (**E**) Liver weight, (**F**) Serum albumin. Data are presented as means ± SEM, *n* = 8, means with different letters differ (*p* < 0.05) according to Tukey’s multiple comparison test. NOM: normal, fed protein-Fe sufficient diet throughout the study, LPI: Low Protein-Fe, STO: *Solanum torvum*, TAD: Cricket + STO, PIS: Protein-Fe sufficient, ADO: Cricket, RFA: Palm weevil larvae.

**Figure 4:**
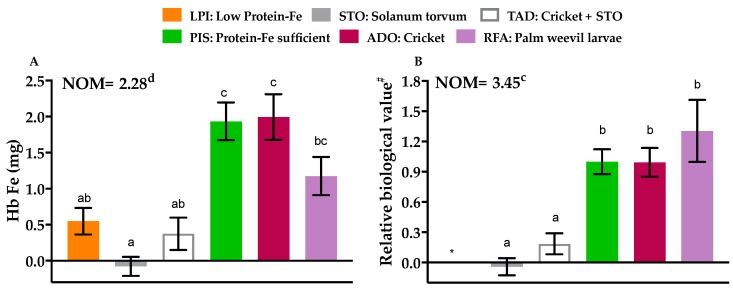
Effects of protein-Fe supplementation on hemoglobin. (**A**) Hemoglobin iron (**B**) Relative biological value (RBV). ^#^ RBV was compared to the PIS group. * RBV too low to calculate. Data are presented as means ± SEM, *n* = 8, means with different letters differ (*p* < 0.05) according to Tukey’s multiple comparison test. NOM: normal, fed protein-Fe sufficient diet throughout the study, LPI: Low Protein-Fe, STO: *Solanum torvum*, TAD: Cricket + STO, PIS: Protein-Fe sufficient, ADO: Cricket, RFA: Palm weevil larvae.

The values for each outcome variable for the NOM group that was never malnourished are provided in the graphs in Figure 3 and Figure 4 and included in the analysis. As expected, change in BW during the 14 day repletion was lower in the LPI (20.1 ± 1.5 g) and STO (10.6 ± 1.7 g) groups, and this was different from the other groups (Figure 3A; *p* < 0.0001). There were no differences in weight gain among the TAD (70.8 ± 4.4 g), PIS (84.6 ± 3.1 g), and RFA (72.6 ± 3.2 g) groups. Weight gain in the ADO (94.3 ± 4.3 g) group was not different from the PIS group but significantly higher than the NOM (70.6 ± 4.4 g) group. The change in LM and BMC were comparable among the TAD, PIS, ADO, and RFA groups (Figure 3A–C). There were no differences in FM between PIS (12.85 ± 0.70 g) and ADO (12.36 ± 1.47 g), between TAD (10.30 ± 1.48 g) and RFA (5.73 ± 1.46 g), and among the LPI (2.64 ± 0.58 g), STO (3.10 ± 0.75 g), and RFA groups. Changes in LM, FM, and BMC were lowest between the LPI and STO groups (Figure 3A–C).

Brain weight in the RFA (0.15 ± 0.02 g) group was significantly higher than in the STO (0.02 ± 0.01 g) and LPI (0.07 ± 0.03 g) group (Figure 3D). There were no differences in brain weight among the TAD (0.13 ± 0.04 g), PIS (0.13 ± 0.02 g), ADO (0.11 ± 0.023g), RFA, and NOM (0.25 ± 0.03 g) groups. Liver weights were similar among the STO (2.09 ± 0.26 g), TAD (2.42 ± 0.26 g), PIS (2.25 ± 0.27 g), ADO (2.80 ± 0.40 g), and RFA (1.83 ± 0.38 g) groups. Spleen and kidney weights were similar among the TAD, PIS, and RFA groups. Overall brain, liver, spleen, and kidney weights were comparable between PIS and ADO and RFA. Also, the liver, kidney, and spleen weights were significantly higher in the NOM group compared to all the other groups (Figure 3D,E). Serum albumin was lower in LPI (0.11 ± 0.01 g/dL) and STO (0.09 ± 0.01 g/dL) but significantly higher in the other groups (Figure 3F; *p* = 0.002). More importantly, there were no significant differences in serum albumin among the TAD (0.31 ± 0.04 g/dL), PIS (0.34 ± 0.02 g/dL), ADO (0.43 ± 0.05 g/dL), RFA (0.29 ± 0.02 g/dL), and NOM (0.33 ± 0.02 g/dL) groups (Figure 3F; *p* = 0.07). 

Hb Fe was not significantly different among the PIS (1.94 ± 0.26 mg), ADO (1.99 ± 0.32 mg) and RFA (1.17 ± 0.27 mg) groups (Figure 4A). Hb Fe in the PIS and ADO groups was significantly different from the LPI, STO, and TAD groups (Figure 4A; *p* < 0.0001). Hb Fe was lowest for the STO (−0.08 ± 0.13 mg) and LPI (0.55 ± 0.18 mg) groups but highest for the NOM (2.28 ± 0.18 mg) group (Figure 4A). Hb Fe was comparable among the LPI, TAD, and RFA groups (Figure 4A). Among all the supplemented groups, the ADO group had the greatest change in Hb Fe, though this was not different from the RFA or PIS groups.

When RBV was calculated based on Hb Fe and food intake, there was an increase in the RBV of the RFA group. In fact, the RBV of either ADO (0.99 ± 0.14) or RFA (1.30 ± 0.31) was as good as that of the PIS (1.00 ± 0.12) group (Figure 4B; *p* = 0.83). RBV was significantly lower for the other test diets but was highest for the NOM (3.45 ± 0.27) group (Figure 4B). 

## 4. Discussion

Edible insects have always been a part of the human diet as sources of protein and micronutrients. Though edible insects are classified as sustainable diets and have been promoted by the FAO since 2013, this is the first animal study to evaluate their protein and Fe bioavailability. Furthermore, this is the first study to use the hemoglobin regeneration efficiency method to examine the Fe bioavailability of the turkey berry. Turkey berry is an underutilized food that is used in many parts of the world, in part, because of its high hematinic and polyphenolic properties [9,12]. For the edible insects, we focused on cricket and palm weevil larvae because of their popularity in both developed and developing countries. Currently, cricket is farmed in the United States and other developed countries, whereas palm weevil larvae farming is underway in developing countries such as Lao People’s Democratic Republic and Ghana. Depending on the commercial farming practices, recipe and portion size, cricket, and palm weevil larvae could provide excellent sources of protein and micronutrients in human diets. The levels of protein in crickets have been reported to be comparable to meat products, but palm weevil larvae has half of the amount [8]. Based on the FAO food composition database, Payne et al. also reported higher iron content of cricket and palm weevil larvae than meat products, and their nutrient value scores were significantly healthier than beef and chicken [8]. 

The use of a protein-Fe deficient rat model mimics the common human protein-Fe deficiency. This rat model of protein-Fe deficiency induced by feeding 5% protein and ~2 ppm iron diet was useful because the body composition, serum albumin, and Hb Fe were higher in the nourished group compared to the malnourished group after the 21 day feeding period. Consistent with previous studies on protein restriction in rats [13,14], we observed a 50% and 95% reduction in food intake and weight gain in the malnourished group. 

During the 14 day repletion period, the protein-Fe deficient rats fed the test protein diets increased their food intake in an attempt to catch-up on BW, LM, and FM relative to NOM rats fed the protein-Fe sufficient diet throughout the study, a finding consistent with a previous refeeding study in rats [13]. The higher protein efficiency of these edible insects may have resulted in increased weight, LM, and FM. These findings have important implications for combating under-nutrition, particularly in developing countries. Contrary to the views expressed by Payne et al. [8], our findings show that crickets and palm weevil larvae are not potential culprits for diet-induced non-communicable diseases (i.e., cardiovascular disease, diabetes, and hypertension) related to over-nutrition. 

Consistent with a previous rat study [15], simple, fast, and noninvasive DXA clearly discriminated against the effects of the LPI diet and protein supplemented diets on body composition. Also, the superior protein and fat content of crickets and palm weevil larvae relative to turkey berry may have accounted for the differences in body composition of the supplemented diets. Given that edible insects are animal-sourced proteins, it is not surprising that the body composition measures of rats fed diets supplemented with edible insects were comparable to the PIS group. However, the values being lower than NOM group that was never malnourished suggests that the duration of repletion might not have been enough to recover completely. The no difference in body composition measures between the LPI and STO group is likely because of the low protein content of STO, which was not enough to reverse the prior malnutrition. However, adding cricket to STO, significantly improved body composition relative to the LPI group. This beneficial effect can largely be attributed to cricket, indicating that turkey berry is not an adequate source of protein. 

In agreement with previous studies [14,16,17], continuous feeding of the LPI diet for the next 14 days decreased brain, liver, and spleen weights compared to the repleted groups. The higher brain weight in the repleted rats may reflect higher brain development and cognitive function similar to the NOM group fed the protein-Fe sufficient diet throughout the study [16]. In contrast, a previous work by Culley and Lineberger [17] showed significantly lower increases in brain weight relative to control rats after refeeding for 110 days following restricted feeding for 60 days. The differences might be due to a longer duration of malnutrition in the previous study. Also, the significant gain in brain weight in this study may be attributed to the high protein and fatty acid content of the edible insects. Specifically, the considerable amount of mono- and polyunsaturated fatty acids in the palm weevil larvae may have been the driving factor in the observed brain weight among repleted rats. 

During malnutrition, protein synthesis by the liver is altered, resulting in low serum albumin levels, a functional measure of protein status. Following 14 day repletion after malnutrition for 21 days, there was a significant increase (not significantly different from NOM group) in serum albumin concentration with insect groups, a finding consistent with previous studies [18,19]. This finding indicates that the protein content and the quality of the protein from the edible insects are adequate to prevent protein malnutrition. 

In addition to the above, animal hemoglobin repletion studies offer a useful and inexpensive measure of bioavailability of iron compounds from foods. However, rats have generally proven to be poor models for human iron absorption as influenced by dietary enhancers and inhibitors [20]. However, in the only published comparison study, Forbes et al. [21] reported that RBV agreed well between the rat hemoglobin repletion method and a human radiotracer method [21]. We modified the HRE method of Forbes et al. [21] by extending the depletion period from 7–8 days to 21 days and the repletion period from 9–10 days to 14 days, and calculated the RBV based on the Hb Fe method [11]. Also, the HRE method focused only on Fe deficiency, but our model included both protein and Fe deficiency. Since Hb Fe in the NOM group was significantly higher compared to all other groups, the 14 day repletion period in our model may not be enough to bring Hb Fe to normal levels and replete Fe stores. 

Although Fe bioavailability from cricket was comparable to the PIS group, an in vitro study [7] showed otherwise. Apart from the differences in methods and the food matrix, the house cricket *A. domesticus* used in this study has variable mineral concentrations compared to the field cricket *G. bimaculatus* used in the previous study. These two crickets are heterogenous species, and edible forms can be obtained at various stages of metamorphosis. Also, evident in the literature are discrepancies in the lack of correlation between in vitro and in vivo bioavailability studies [22]. Notably, the primary source of heme iron in crickets and palm weevil larvae may be in cytochromes, and we presume that its bioavailability is comparable to the heme iron of hemoglobin. Insects also have iron bound to the non-heme molecules, ferritin and holoferritin [23]. Fe associated with these proteins is typically in the ferrous state, which may have high bioavailability. 

From our study, it is unclear whether the improvements in RBV with palm weevil larvae might be due to enhancing factors in the larvae. Considering the low Fe content of the larvae, our dietary data suggests that the high-fat content of the larvae, may have improved its RBV. In an Fe-deficient rat model, diets high in saturated or unsaturated fats had an enhancing effect on Fe absorption [24]. Proximate analysis of the larvae indicates an unsaturated/saturated fatty acid ratio of 1:11 [25]. Consistent with previous studies [24,26,27], we speculate that the high saturated fatty acid content of the larvae may have resulted in higher hemoglobin regeneration. Possible means by which fat might influence iron absorption include stimulating bile acid secretion to favor Fe absorption or forming fatty acid-Fe complexes in the lumen that helps to maintain Fe in solution and favors its absorption [28,29]. 

Though the Fe content of turkey berry is high [9], the Hb-Fe repletion was very low in our study. Contrary to our findings, an aqueous extract of turkey berry increased Hb concentration in a dose-dependent manner in phenylhydrazine-induced anemic rats [12]. It is difficult to compare our results with Koffuor et al. [12] due to differences in the study design and method of inducing anemia. In phenylhydrazine-induced anemia [12], free radicals are formed, causing hemolysis of red blood cells (RBCs) resulting in tissue hypoxia. Since turkey berry is a potent antioxidant [30], it may have quenched the free radicals. It is important to consider that tissue hypoxia stimulates erythropoietin production, regulating the proliferation and differentiation of hematopoietic progenitor cells in the bone marrow to reverse the anemia [31]. In this study, the low protein content of the turkey berry coupled with protein deficiency in the rats may have reduced erythropoietin production resulting in low Hb. However, increasing the protein content by adding crickets to turkey berry resulted in only slight increases in Hb Fe, which might be explained by the considerable amounts of Fe absorption inhibitors such as polyphenols and phytic acid in turkey berry [30]. Cooking for a short time and removing its seed coat as well as storage overnight or for a few days may be beneficial in lowering the polyphenolic and phytic acid content whiles preserving its antioxidant properties [32]. 

It is important to note that very few human studies examining the effects of insect consumption on nutritional outcomes exist. Though no human studies have been conducted in children using crickets, a study with adults showed cricket consumption is tolerable, may improve gut health, and reduce systemic inflammation [33]. For two studies in which children were fed a caterpillar cereal [34] and termite cereal [35], there were no differences in growth, but positive effects were seen on Hb levels compared with control groups. Although animal studies may not always translate into humans, the findings of this study support that cricket and palm weevil larvae could be alternative protein and iron sources and provide a basis to design future human studies, particularly among children. Our findings contribute to current efforts in the search of novel food alternatives to combat protein and Fe deficiency. In countries where edible insects are culturally accepted, they could be novel food additives for commercial or home fortification. 

## 5. Conclusions

Interestingly, turkey berry has a high amount of iron, but poor Fe bioavailability, maybe due to its high polyphenol and phytic acid content. Our study showed that cricket and palm weevil larvae are excellent sources of protein and bioavailable iron since improvements in protein status and Hb Fe was similar to high-quality protein (casein) and highly bioavailable Fe (FeSO_4_), respectively. Cricket and palm weevil larvae powders could provide significant proportions of daily recommendations of protein and minerals in the diets of humans. In countries where these edible insects are culturally accepted as food, they could provide a low-cost sustainable and alternative source of protein and bioavailable iron to prevent protein and iron deficiency in children. 

## Figures and Tables

**Table 1:** Composition of test diets.

	Test Diets (g/kg) ^#^					
	Low Protein-Fe [LPI]	*S. torvum* [STO]	*A. domesticus* + *S. torvum* [TAD]	Protein-Fe Sufficient [PIS]	*A. domesticus* [ADO]	*R. phoenicis fabricius* [RFA]
Ingredients						
Casein, low Cu and Fe	57.5	57.5	37.4	172.4	57.5	57.5
Corn starch	559.5	250.7	311.4	449.2	321.6	233.2
Sucrose	100.0	100.0	100.0	100.0	100.0	100.0
Soybean oil	70.0	117.0	70.0	68.8	33.3	0.0
Mineral mix, Fe deficient	35.0	35.0	35.0	35.0	35.0	35.0
Vitamin mix, AIN-93-VX	10.0	10.0	10.0	10.0	10.0	10.0
Ferrous Sulfate, heptahydrate	0.0	0.0	0.0	0.1	0.0	0.0
Choline Bitartrate	2.5	2.5	2.5	2.5	2.5	2.5
*S. torvum*	0.0	267.0	121.3	0.0	0.0	0.0
*A. domesticus*	0.0	0.0	154.3	0.0	283.0	0.0
*R. fabricius*	0.0	0.0	0.0	0.0	0.0	403.0
Nutrients						
Energy (kcal/g)	3.9	3.9	3.9	3.9	3.9	4.3
Protein (%)	5.0	8.8	15.0	15.0	23.3	15.0
Carbohydrate (%)	76.8	60.9	59.9	66.9	55.4	57.8
Fat (%)	7.1	12.4	10.2	7.1	8.6	15.8
Iron (ppm)	0.0	20.0	20.0	20.0	20.0	10.0
Zinc (ppm)	35.6	43.1	68.9	35.6	90.5	112.1

^#^ Abbreviations of the test diet are indicated in the square bracket.

**Table 2:** Comparison of food and nutrient intake during 21 day feeding in baseline rats ^1^.

	Test Diets		
Nutrient Intake	Malnourished	Nourished	*p*-Value *
Food intake (g)	164.2 ± 14.5	313.9 ± 10.1	<0.0001
Energy (kcal)	640.4 ± 56.6	1224.4 ± 39.4	<0.0001
Protein (g)	8.2 ± 0.7	47.1 ± 1.5	<0.0001
Carbohydrates (g)	126.1 ± 11.2	210.0 ± 6.8	0.002
Fats (g)	12.5 ± 1.1	22.1 ± 0.7	<0.0001
Iron (mg)	0.0 ± 0.0	6.3 ± 0.2	<0.0001
Zinc (mg)	5.9 ± 0.5	11.2 ± 0.4	<0.0001

^1^ Values are means ± SEM, *n* = 5, * Significant at *p* < 0.05 according to students’ *t*-test.

**Table 3:** Food and nutrient intake of rats during the 14 day repletion period ^1^

	Test Diets ^2^						
Nutrient Intake	NOM	LPI	STO	TAD	PIS	ADO	RFA
Food intake (g)	281.9 ± 12.4 ^d^	147.7 ± 5.4 ^a^	201.3 ± 11.5 ^b^	246.7 ± 11.3 ^cd^	225.6 ± 6.0 ^bc^	227.2 ± 10.5 ^bc^	212.8 ± 8.2 ^b^^c^
Energy (kcal)	1099.4 ± 48.4 ^d^	576.0 ± 21.0 ^a^	784.9 ± 44.6 ^b^	962.1 ± 43.9 ^cd^	879.6 ± 23.2 ^bc^	886.2 ± 41.1 ^bc^	915.2 ± 35.4 ^bc^
Carbohydrates (g)	42.3 ± 1.9 ^c^	7.4 ± 0.3 ^a^	30.2 ± 1.7 ^b^	37.0 ± 1.7 ^bc^	33.8 ± 0.9 ^b^	52.9 ± 2.5 ^d^	31.9 ± 1.2 ^b^
Protein (g)	188.6 ± 8.3 ^d^	113.4 ± 4.1 ^a^	134.6 ± 7.7 ^abc^	147.8 ± 6.7 ^bc^	150.9 ± 0.4 ^c^	125.9 ± 5.8 ^abc^	123.0 ± 4.8 ^ab^
Fats (g)	19.9 ± 0.9 ^d^	10.4 ± 0.4 ^a^	14.2 ± 0.8 ^ab^	25.2 ± 1.2 ^e^	15.9 ± 0.4 ^bc^	19.5 ± 0.9 ^cd^	33.6 ± 1.3 ^f^
Iron (mg)	5.6 ± 0.3 ^e^	0.0 ± 0.0 ^a^	4.0 ± 0.2 ^c^	4.9 ± 0.2 ^de^	4.5 ± 0.1 ^cd^	4.5 ± 0.2 ^cd^	2.1 ± 0.1 ^b^
Zinc (mg)	10.0 ± 0.4 ^b^	8.7 ± 0.5 ^b^	17.0 ± 0.8 ^c^	8.0 ± 0.2 ^ab^	20.6 ± 1.0 ^d^	23.9 ± 0.9 ^e^	10.0 ± 0.4 ^b^

^1^ Values are means ± SEM, *n* = 8. Within a row, means with different superscripts differ (*p* < 0.05) according to Tukey’s multiple comparison test. ^2^ Test diets: NOM = normal, fed protein-Fe sufficient diet throughout the study, LPI = Low protein-Fe, STO = *Solanum torvum*, TAD = Cricket + STO, PIS = Protein-Fe sufficient, ADO = Cricket, RFA = Palm weevil larvae.
